# On Sedative Efficiency

**Published:** 1802-06

**Authors:** Robert Kinglake

**Affiliations:** Chilton, super Polden


### *On Sedative Efficiency; communicated by* Dr. Kinglake.

NO queftion in Phyfiology has been lefs fatisfactorily agitated than that which divides the medical world on the operation of fedative fubftances. The prevailing opinion, however, feems to be the leaft defenfible, and to have been adopted much too gratuitoufly to be entitled to admiffion.

It is familiarly imagined, that certain fubftances act on the animal economy as direct fedatives, that is, that they reprefs the action of motive power; but thofe who entertain this opinion do not feel equal facility, in affigning a reafon for this extraordinary effect. It is roundly afferted, that inordinate action is diminifhed by fome power in the fedative agent, capable of fubduing the exifting motive power.

The falutary as well as morbid actions of life, may be undoubtedly altered by the influence of medicinal fubftances; but no fubftance is capable of directly retarding vital action by inducing debilitating changes in the motive conditions of life; on the contrary, fuch atonic changes would tend rather to generate difeafe, than to ferve as a remedy, as is evinced by all diforders of debility being characterifed by more or lefs of accelerated and irregular motion.

It muft be evident to common obfervation, that diminifhed motive power is infeparably connected with proportionate celerity of action; this is ftrikingly exemplified in the latter ftages of acute, and in the ufual progrefs of chronic difeafes, particularly thofe in which nervous affection predominates. The doctrine therefore of fubftances operating fedatively, by debilitating or directly abftracting motive power, feems wholly untenable.

Sedative influence may obtain in two different ways, either by univerfally exciting and invigorating the fyftem, or by withholding or prohibiting exceffive ftimulation; in the former order of efficiency may be ranked whatever may be capable of fubduing, by congenial excitement, morbid debility and its confequent quickened action; in the latter may be included a fuitably reduced temperature, abftinence, increafed evacuations, fhaded light, and mental depreffion.

The mode in which thefe pofitive and negative powers operate, is formally oppofite but efficiently fimilar; the one retards hurried action by the fuperceding influence of acceflorial vigour, while the other obviates undue exhauftion by withholding noxious excitement.

Thefe different fpecies of fedative power are applicable to oppofite ftates of difeafe; the pofitive influence is adapted to reftrain restrain and controul the quickened action of debility, while the negative is suitable to diminishing immoderate excitement.

If, then, morbidly accelerated action, in all cases of debility, originates from a weakened state of vital power, and can only be retarded and invigorated by imparting additional force to the reduced power, it is obvious, that effects positively sedative can only be sought for in the use of the most efficient narcotics, tonics, and stimulants. Thus experience confirms, that quickened action, whether particularly agitating the arterial or nervous systems, is most effectually repressed by opium, digitalis, hyoscyamus niger, cicuta, belladonna, nicotiana, cinchona, cascarilla, angustura, ferrum, zincum, cuprum, alumen, ammonia, æther, camphora, moschus, valeriana, asafœtida, &c. and above all, by pure air, small quantities of nutriment at short intervals, with suitable exercise, tranquillity, and sleep. In proportion as these agents operate salutarily, the diseased celerity of action will be retarded, and in the exact ratio of returning slowness will be the increased firmness of the motion. In the action of the arteries this improved vigour will be denoted by augmented fulness and softness of the pulse; in the nervous system by steady and commanding sensations; in the muscular structure by firm and unwearied exertion.

When vascular plenitude, distension, and the re-active violence of comparatively unimpaired tone induce either local inflammation, or acute general disease, the excessive action demands the most efficient influence of sedative power; but the effect under these circumstances can only be rendered negative by abstracting or diminishing the force of the stimulant powers before specified.

It is of the last importance rightly to discriminate the mode of sedative operation, that effectual means may be occasionally resorted to for its accomplishment. In the common acceptation of sedative power, a debilitating plan of treatment would be universally held to be conducive to its best effects; but which indeed would tend to increase the cause of the disorder requiring real sedative aid, and would probably render it too inveterate to be afterwards overcome by the most appropriate means of cure; on the other hand, in these cases of vigorous action demanding the negative effect of sedative power, agents possessing positive efficiency might be employed, such as opium, digitalis, hyoscyamus, nicotiana, &c. to the manifest increase of the disease, by causing additional excitement.

The effects which digitalis produce in the vascular system, have been held as an unquestionable proof of its debilitating operation. It is true, that its influence renders a rapid pulse flow; but it is equally true that this effect is never produced without a proportionate increafe of tone and fulnefs being afforded to the pulfations. This change proves, that what has been loft in celerity has been gained in ftrength, and that the agent producing the effect has augmented, and not diminifhed vital energy.

The effect of digitalis, when it operates falutarily, (that is, when its influence equally pervades the fyftem, imparting congenial excitement approaching to the healthful ftimulation of natural powers) is to retard rapid action, and to obviate undue excitement by equilibrating and energifing vital power.

No difficulty is experienced in admitting that want of tone in the fyftem is defignated by inordinately quick and irregular action; yet, by a ftrange perverfion of reafon, it is fuppofed that this atonic effect may be refifted by increafing the exifting debility. The retarding and tranquilifing influence of digitalis has been thus explained even in oppofition to the ftrong teftimony to the contrary, afforded by its tonic effects in phthifical, hydropic, and hæmorrhagic affections. In thefe cafes, tottering feeblenefs marks the advanced degree of prevailing debility, as evidently as the accelerated and weakened ftate of arterial action. Would not increafed debility, under thefe circumftances, aggravate the difeafe? And if digitalis acted as a debilitant, would it not fpeedily annihilate the toil-worn relics of life?

The theory that would contend for digitalis and other narcotic agents operating curatively in difeafes of debility, by fuperadding to the exifting weaknefs, meets an infuperable difficulty in affigning to them at once a debilitating effect on the arterial fyftem, and an excitant influence on the lymphatic ftructure.

How fuch oppofite effects can arife from the fame power, on different parts of the animal body, does not appear, nor does any principle of philofophising, applicable to the animal economy, warrant fuch a contradictory inference; it has therefore been unfcientifically affumed to fubferve the delufion of a pre-conceived erroneous opinion.

What medicinal agent in other cafes has been even fuppofed fimultaneoufly to brace one part of the animal frame, and to relax another? Do cathartic fubftances debilitate the upper and invigorate the lower portion of the inteftinal canal? Do diuretics weaken the fecerning veffels of the kidneys, and ftrengthen the excreting tubes? Or do nervous ftimulants impair the energy of the brain, and impart additional tone to its feveral diftributions over the fyftem? When thefe incongruous effects happen, then may it be admitted that digitalis, and other narcotic powers, powers, diminiſh the action of the arterial ſyſtem, and increaſe that of the lymphatic ſtructure; or that ſedatives repreſs the precipitate motion of debility, by leſſening active energy.

It has not yet occurred to me to witneſs any inſtance of the ſalutary effects of digitalis, in which it did not evidently act as a narcotic ſtimulant, in which opium, hyoſcyamus, and the various tonic and ſtimulant ſubſtances in medicinal uſe, would not have operated ſomewhat ſimilarly.

Opium has indeed, in ſome phthiſical caſes under my obſervation, proved a uſeful auxiliary, and occaſionally, when nauſea has inſeparably accompanied the employ of digitalis, an efficacious ſubſtitute.

Whatever equally excites, and ſuſtains the native energies of the ſyſtem, will retard, ſoften, and fill the pulſe. A moderate quantity of Madeira wine, alimentary refreſhment, increaſed temperature, (particularly that of the warm bath) and pleaſing intelligence, will reſtrain and invigorate the rapid motion of a debilitated pulſe, as ſenſibly, though perhaps not ſo extenſively and durably, as digitalis.

It has not happened to me to obſerve that any ſtrengthening or beneficial effect has reſulted from the employ of digitalis when it has induced diſtreſſing nauſea. This effect is highly debilitating to the ſtomach, and ſympathetically ſo to the ſyſtem at large. The degree of ſickneſs produced, may be accurately meaſured by the increaſed weakneſs and celerity of the pulſe, and its progreſſive retardation and fulneſs will as correctly mark the ſubſidence of nauſea. Certain conſtitutional idioſyncracies render the impreſſion of digitalis wholly intolerable; inſtead of augmenting and equaliſing ſyſtematic energy, it embarraſſes and diſorders every movement. Similar effects ſometimes ariſe from other ſtimulants, particularly from thoſe of the narcotic claſs, and the exceſſive influence of all of them is often formidably deleterious.

Were it poſſible accurately to meaſure the preciſe degree of narcotic influence neceſſary to ſubdue morbid quickneſs of motion, curative effects might be confidently expected from its uſe in a vaſt majority of diſeaſes; but inſtead of this certainty, the ſtimulant operation often unaccountably exceeds the ſtandard of healthy excitement, and involves the powers of life in the equally irreparable difficulties either of exceſſive exhauſtion, or of organic decompoſition.

The deſirable effect of narcotic ſubſtances is to alleviate morbid irritability and ſenſibility, by energiſing, and equally diſtributing the vital motion. Its ſoporific operation marks the exceſs of its ſtimulant power on the brain, and indicates the propriety of diminiſhing its force, leſt by inducing a high degree degree of indirect debility, its tonic influence ſhould be counteracted.

Digitalis remarkably debiliates when it ſtupifies: It ſtrengthens only when it leſſens morbid ſenſation, by moderately invigorating and retarding arterial action. Its ſtimulant effect (like that of all other poſitive ſedatives) is inſufferably noxious in the early ſtage of acute fever, particularly under thoſe circumſtances of general irritation that prevail in viſceral inflammation. It was lately given under my direction, on about the fourth day of a violent peripneumonia, accompanied with a rapid, hard, and contracted pulſe, and much depreſſion of ſtrength. Though the doſe was reſtricted to one grain at intervals of four hours, yet the third exhibition of this ſmall quantity ſeemed to be diſtinctly followed by threatening delirium. Much additional difficulty aroſe from this medicinal interference of hurtful power. The patient ſtruggled, alternately comatoſe and furious, during three days and nights, from which ſtate bliſtering and bleeding appeared at length to have relieved him.

On the eighth day, much ſyſtematic debility and morbid irritability of the lungs prevailed, with laborious reſpiration, an irregular, extremely feeble, and rapid pulſe; ſo much ſo indeed as to be at intervals either countleſs, or wholly imperceptible. The digitalis was now reſumed, one grain of the powder was given every four hours; the effect was all that could be deſired, after a few doſes of the medicine the pulſe filled, ſoftened, and became ſlower, every morbid ſymptom abated; the medicine was continued about a week, during which time progreſſive amendment inſuring ultimate recovery, induced a neglect of its farther uſe.

Another caſe ſince occurred of peripneumonic affection, which did not come under my care until the ſeventh day of its progreſs. Much ſyſtematic debility and pulmonic irritation prevailed, with almoſt inceſſant cough, difficult reſpiration, dry heated ſkin, and a weak, rapid, hard pulſe. The tincture of digitalis was directed in doſes of fifteen drops every four hours; on the ſecond day the ſymptoms were alleviated. The doſe was then extended to twenty drops at the ſame intervals; on the third day the pulſe became ſenſibly more ſoft, full, and ſlow; the urgent cough and embarraſſed reſpiration were much relieved; when a ſalutary determination on the cuticular exhalants at once moiſtened and cooled the ſurface. No obſtacle happened to the courſe of convaleſcency, which ſoon terminated in perfect recovery.

My motive for troubling you with theſe extenſive remarks on the effects of digitalis, is to oppoſe the prevailing opinion of this this agent operating as a *debilitant* ſedative. My attention has been more particularly invited to this ſubject, by obſerving in a late Number* of your Journal, ſome animadverſions offered by one of your very intelligent correſpondents on the ſedative effect of Digitalis. The author alluded to has quoted a note to a former paper of mine, in which my objection to the theory of abſorption is generally made. My opinion of the lymphatic veſſels being paſſive tubes, deſigned to tranſmit the fluid forced into their permanently open orifices by arterial and muſcular impulſe, is oppoſed by the authority cited, by the known effect of digitalis in removing ſerous fluids from cavities under an apparently diminiſhed arterial and muſcular power. On the ſlowneſs and celerity of the pulſe muſt the diſpute be at iſſue. The queſtion then is, whether the weak, ſwift pulſe, denotes more ſyſtematic energy than the comparatively ſlow, full pulſe, which reſults from the beneficial employ of digitalis? The arterial action in a ſtate of health, and the approaches made towards it in all caſes of morbid debility, under the recruitng and ſtimulating effects of alimentary and tonic aid, would ſeem to prove that a ſlow, full pulſe, is founded on more ſtrength than that which is weak and rapid, and therefore that digitalis is a *stimulant*, and not a *debilitant* ſedative; that it invigorates the active powers of the ſyſtem, that it conſequently augments the arterial and muſcular impulſe, and that thereby may be explained its efficacy in removing ſerous effuſions by forcing them into the lymphatic circulation.

In another publication,† my reaſons for believing in *non-absorption* and the paſſively tranſmitting office of the lymphatics are given to ſome extent. It may be here ſufficient to obſerve, that the ſtructure of the lymphatic tube is deſtitute of every requiſite for active exertion, while it is provided with every convenience for admitting and facilitating the paſſage of the lymphatic fluid; that it is ſo extremely ſlender in its fabric as to be diaphanous; that its cavity is highly poliſhed, and ſtudded at ſhort diſtances with valvular obſtacles to refluence; that it has no apparent muſcularity; that it is inſuſceptible of contraction from either mechanical or chemical ſtimulants; that the latter (more particularly ſuch as the mineral acids) may decompoſe and corrugate its ſubſtance, but in no inſtance have they given ſatisfactory proof of muſcular action; that its orifice is elaſtic, permanently open, and inirritable to any exciting agents; that

* See Medical and Phyſical Journal, No. XXXVII. p. 197.

† See Caſes and Obſervations on the Medicinal Effects of Digitalis Purpurea in Phthiſis Pulmonalis, &c. by Robert Kinglake, M. D.

that its texture is too ſlight to endure the ſhock of active abſorption, and propelling contraction; and, finally, that its paſſively tranſmitting function is equally verified by the phenomena of health and diſeaſe. The impulſive and propulſive powers in the healthful ſtate of the animal œconomy, inſure a regular refluence of lymph.

In the acute periods of either febrile, or other violent affections, the lymphatic circulation is ſo precipitated, as to cauſe a deficiency of exhalant fluid in the cellular and interſtitial cavities; while in chronic maladies, a want of adequate circulating power is apt to occaſion a redundance, and an accumulation of it. In the one caſe, debilitating or negatively ſedative powers would tend duly to moiſten the cavities; in the other, additional ſtimulant power (ſuch as Digitalis and other analagous agents eminently afford) would force into the lymphatic circulation the exhaled and ſtagnant fluid.

*Chilton, ſuper Polden,*

*May* 14, 1802.

ROBERT KINGLAKE.

